# Ocular adverse events from pharmacological treatment in patients with multiple sclerosis—A systematic review of the literature

**DOI:** 10.1186/s13643-021-01782-7

**Published:** 2021-10-28

**Authors:** Juliana Muñoz-Ortiz, Juliana Reyes-Guanes, Estefanía Zapata-Bravo, Laura Mora-Muñoz, Juan Antonio Reyes-Hurtado, Luis Octavio Tierradentro-García, William Rojas-Carabali, Marcela Gómez-Suarez, Alejandra de-la-Torre

**Affiliations:** 1 Neuroscience Research Group “NeURos”, Escuela de Medicina y Ciencias de la Salud, Universidad del Rosario, Carrera 24 # 63C – 69, Bogotá, Colombia; 2 Escuela Barraquer Research Group, Escuela Superior de Oftalmología del Instituto Barraquer de América, Avenida Calle 100 # 18A - 51, Bogotá, Colombia

**Keywords:** Drug-Related Side Effects and Adverse Reactions, Multiple Sclerosis, Therapeutics, Eye Diseases

## Abstract

**Purpose:**

The aim of this study was to review the scientific evidence and describe the ocular treatment-emergent adverse events (TEAEs) related to pharmacological treatment in patients with multiple sclerosis.

**Methods:**

A systematic review of literature was conducted according to the Preferred Reporting Items for Systematic Reviews and Meta-analysis guidelines in the MEDLINE, LILACS, EMBASE, and COCHRANE databases. Articles were filtered based on title and abstract considering the selection criteria and subsequently filtered by full-text reading. The resulting articles were evaluated using the Joanna Briggs Institute Quality Tools. Study characteristics and results were extracted and presented in structured tables to conduct a narrative synthesis.

**Results:**

A total of 2852 published articles were extracted using our strategy. After removing duplicates, 2841 articles were screened based on title and abstract, 102 articles were evaluated using quality tools, and 69 articles were filtered by full-text reading. Through this search strategy, 60 articles met all the inclusion criteria and seven articles, through a search update conducted in the same manner, were included. This resulted in 67 articles meeting the inclusion criteria, of which 11 were experimental and 56 were observational. The therapies related to ocular TEAEs were alemtuzumab, amantadine, fingolimod, steroids, CTLA-4 Ig, estriol, interferon β, natalizumab, hyperbaric oxygen, rituximab, siponimod, teriflunomide, and tovaxin. Fingolimod and siponimod were commonly associated with macular edema, interferon β was associated with retinopathy, alemtuzumab was associated with thyroid eye disease, amantadine was associated with corneal edema, and steroids were associated with acute retinal necrosis. Opportunistic infections were also found, and there was one life-threatening case.

**Conclusions:**

Our search revealed different methodological assessments of the topic. However, longitudinal studies regarding ocular TEAEs related to multiple sclerosis therapy are necessary to provide evidence-based recommendations, especially in understudied regions such as Latin America and Africa. Physicians should monitor ocular symptoms in patients being treated for multiple sclerosis and consider an interdisciplinary approach.

**Systematic review registration:**

PROSPERO ID CRD42020106886

**Supplementary Information:**

The online version contains supplementary material available at 10.1186/s13643-021-01782-7.

## Key points

- This study was conducted to review the scientific evidence and describe the ocular TEAEs related to pharmacological treatment in patients with multiple sclerosis to provide a theoretical basis and quality literature for ophthalmology specialists, neurologists, and general practitioners, which could help them in making appropriate clinical decisions.
- Patients under fingolimod and interferon β treatment showed a higher frequency of TEAEs. Alemtuzumab was associated with an autoimmune response that manifested as thyroid eye disease.
- We recommend physicians to be cautious when treating patients with multiple sclerosis and monitor the ocular symptoms that patients may present. An interdisciplinary approach might be considered to evaluate the patient’s requirements.

## Background

Multiple sclerosis (MS) is a chronic autoimmune demyelinating disease with variable clinical, radiological, and pathological characteristics. Since its first description by Charcot, there have been significant efforts to clarify its pathophysiology and progression and determine the best strategies for appropriate management [1]. Currently, the therapy for MS comprises corticosteroids, immunosuppressants, and immunomodulators such as interferon (INF) and monoclonal antibodies. Although several cases of patients with ocular TEAEs have been reported over the years, there is a lack of clear understanding of the TEAEs that are strictly related to the disease and linked only to therapy [2].

Ocular manifestations in patients with MS are not rare and have been described as a major aspect of the disease. Any structure related to visual pathways could be involved in MS, and up to 20% of patients would display optic neuritis as an initial clinical manifestation. Visual field and color vision defects, relative afferent pupillary defect, and other ophthalmological findings could also be present [3].

According to the World Health Organization (WHO), the definition of drug-related adverse events corresponds to an involuntary harmful response to a medication used in a normal dose for a prophylactic, diagnostic, or therapeutic purpose [4, 5]. However, it is necessary to consider that this harmful response, irrespective of how minor it is, must be explained to the patient before initiating any medication to predict, prevent, or treat any possible future event and avoid poor adherence to treatment [5]. Some of the TEAEs that have been described as related to therapy in MS are cataract, glaucoma, chorioretinopathy, visual acuity reduction, macular edema, retinopathy, among others [2].

The aim of this study was to review the scientific evidence and describe the ocular TEAEs related to pharmacological treatment in patients with MS to provide a theoretical basis and quality literature for ophthalmology specialists, neurologists, and general practitioners, which could help them in making appropriate clinical decisions.

### Methods

This review was written according to the Preferred Reporting Items for Systematic Reviews and Meta-analysis (PRISMA) extension statement for reporting systematic reviews. The protocol registration can be found under the PROSPERO ID CRD42020106886.

### Study design

A systematic literature review focusing on ocular TEAEs in MS therapy was conducted using the MEDLINE, LILACS, EMBASE, and COCHRANE databases for articles published till November 2018. The search was updated on May 4, 2020. MEDLINE and COCHRANE databases were searched using Medical Subject Headings terms, EMBASE was searched using emtree terms, and LILACS was searched using Descriptores en Ciencias de la Salud terms, using boolean operators, as evidenced in Annex 1. We limited the search only to human studies, although no limits regarding language and period of publication were used.

### Study selection

An initial search was conducted by JMO, JRG, LMM, and JARH, based on which a review was prepared to eliminate duplicates. Eligible studies were selected by screening the title and abstract by at least two reviewers independently, and discrepancies were resolved by an expert evaluator (ADLT). A study was included if (a) the abstract was available, (b) it contained original data, (c) the diagnosis of MS was made by a specialist, (d) the individual was being treated for MS, and (e) the ocular manifestations appeared after the initiation of MS treatment. Experimental and observational studies were also included. Articles were excluded from the analysis if they did not provide information regarding ocular TEAEs in individuals undergoing MS therapy.

The full texts of the selected articles were retrieved and classified according to the type of article/study by at least two reviewers independently, and discrepancies were resolved by an expert evaluator (MGS). They were then evaluated using the Joanna Briggs Institute Quality Tools (JBIQT). If an article was not available, contact with the author was made. The cascade of article selection was managed using the PRISMA flowchart [6]. The following information was extracted from the selected articles: study period, location, number of patients, type of medication, and ocular TEAEs. A dataset was constructed using the information mentioned ahead.

### Data synthesis and presentation of results

Microsoft Excel (Microsoft Corp., Redmond, WA, USA) was used as a synthesis tool to organize the results of the search strategy and the information of the articles. The first sheet contained information about the initial search results without duplicates. The next six sheets were asigned to three pairs of authors (LMM vs JARH, WRC vs JRG, and JMO vs LOTG) to filter titles and abstracts in a peered manner. A color code was used to select the articles as follows: green to include, yellow to revise, and red to exclude. The next three sheets were used to evaluate concordance for each pair of authors. The subsequent sheet was used by the expert reviewer (ADLT) to resolve discrepancies from the peered review. An additional sheet was used to classify the methodological design of the studies and record JBIQT grades and the reasons for exclusion. The next sheet was used to record articles included after full-text reading. The final sheet included information regarding ID number, authors, year of application of the study, year of publication, journal, location, title, aim, sample size, population (gender and group characteristics), methodological design, MS type, therapy, time of presentation of TEAEs, symptoms after therapy suspension, statistical analysis, important details from results, important details from discussion, and observations. The same synthesis tool was used for the search strategy update. The extracted data were synthetized in tables and figures to present them in an organized manner throughout the article.

### Risk of bias

We retrieved and classified the articles based on study design to evaluate their methodological quality using their respective JBIQT [7]. Each quality tool has a set of questions that are evaluated as *yes*, *no*, *unclear*, or *not applicable* domains*.* An expert in epidemiology (MGS) established a minimum cut-off point as the number of items on the tool marked as “Yes,” including the major and minor criteria. Case reports with 5/8 items on the checklist were included; for case series, the minimum cut-off point was 6/10 items; for cross-sectional studies, it was 6/8; for randomized controlled trials (RCTs), it was 10/13; and for nonrandomized experimental studies, it was 6/9 (see Annex 2 for supplementary information).

## Results

### General description

**Fig. 1:**
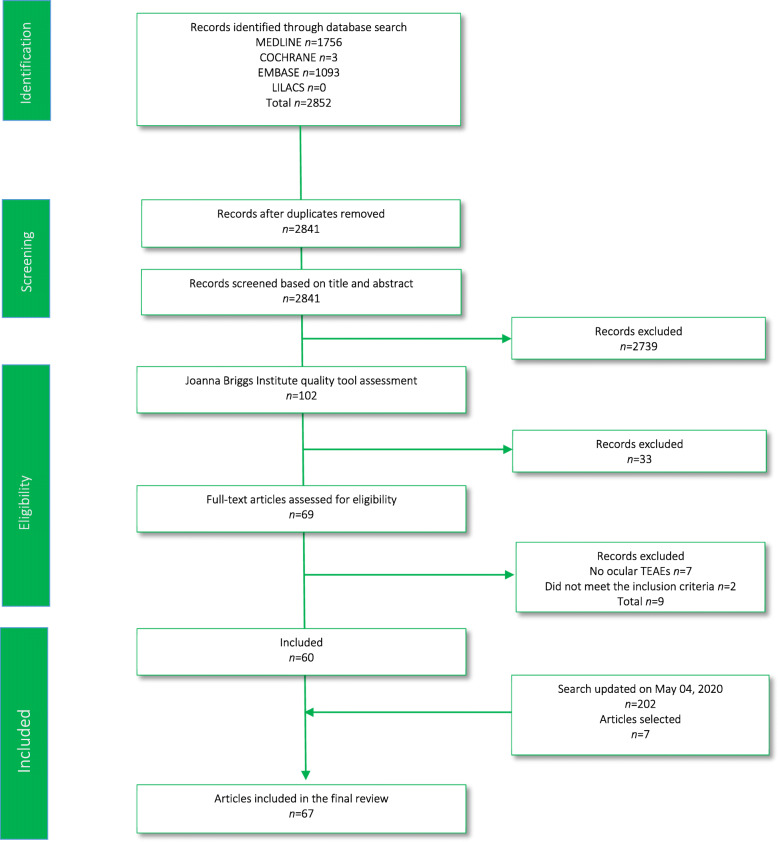
PRISMA flowchart

We extracted 2852 published articles (1756 from MEDLINE, 1093 from EMBASE, three from COCHRANE, and none from LILACS). After removing duplicates, 2841 articles were screened based on title and abstract, and 102 articles were evaluated using JBIQT. After quality evaluation, 33 articles were excluded, and 69 full-text articles were assessed for eligibility. Sixty articles met all the inclusion criteria. Figure 1 shows further detailed information regarding the inclusion of articles**.**

The search was updated on May 04, 2020, extracting 202 published articles (117 from MEDLINE, 85 from EMBASE, none from COCHRANE, and none from LILACS). After removing duplicates, 196 articles were screened based on title and abstract. A total of 11 articles were evaluated using JBIQT. After quality evaluation, two articles were discarded, and nine full-text articles were assessed for eligibility. Finally, after discarding two articles, as no ocular TEAEs were reported, seven articles met all the inclusion criteria (Fig. 1).

### Characteristics

Our final inclusion strategy yielded 67 published articles, including 20 in the United States of America (USA), 24 in Europe (Germany, Spain, France, Italy, Wales, England, Switzerland, Croatia, and Sweden), three in Canada, three in Australia, nine in Asia (Japan, Israel, Iran, and Turkey), and eight were multicentric across continents.

All studies were published between 1987 and 2020. Most of the studies included only adults, but one retrospective cross-sectional study included adults and children [8]. Of the 67 studies, 38 were case reports, four were case series, 14 were cross-sectional studies, and 11 were experimental studies. Table 1 summarizes the characteristics of the studies included in the present systematic review.

**Table 1:** Characteristics of studies

Author	Year	Study design	Country	Sex	Patients with TEAEs	TEAE	Post-TEAE
ALEMTUZUMAB							
Tsourdi et al [59]	2015	Case series	Germany	Both	2/5	Thyroid eye disease	Definitive treatment still discussed / Near-total thyroidectomy and 100 μg levothyroxine per day
Trinh et al [53]	2015	Case report	USA	Female	1/1	Thyroid eye disease	Improvement after thyroidectomy and conservative management
Roos et al [61]	2018	Case series	England	Both	6/162	Thyroid eye disease	Three patients required systemic immunosuppression and three were managed conservatively
Tuohy et al [70]	2014	Cross-sectional	England	Both	2/87	Herpes zoster ophthalmicus	Not reported
Willis et al [72]	2016	Cross-sectional	Wales and England	Both	1/100	Conjunctivitis	Not reported
AMANTADINE							
Jeng et al [35]	2008	Case report	USA	Both	3/3	Corneal edema	Improvement after treatment withdrawal in two patients. One patient required penetrating keratoplasty
Esquenazi [30]	2009	Case report	USA	Female	1/1	Corneal edema	Improvement after treatment withdrawal and topical prednisolone 1%
FINGOLIMOD							
Calabresi et al [9]	2014	RCT	Multicentric	Both	9/1083	Macular edema	Improvement after treatment withdrawal
Cohen et al [10]	2010	RCT	Multicentric	Both	6/1292	Macular edema	Improvement after treatment withdrawal
Akiyama et al [21]	2016	Case report	Japan	Female	1/1	Macular edema	Patient refused treatment withdrawal and macular edema resolved
Chui et al [27]	2013	Case report	Australia	Female	1/1	Macular edema	Patient refused treatment withdrawal and improvement was observed after treatment with ketorolac and dexamethasone
Jasani et al [34]	2017	Case report	England	Female	1/1	Macular edema	Improvement after treatment withdrawal
Kim et al [37]	2015	Case report	USA	Female	1/1	Macular edema	Improvement after treatment withdrawal, topical ketorolac and topical prednisolone
Minuk et al [41]	2013	Case report	USA	Female	1/1	Macular edema	Patient refused treatment withdrawal and improvement was observed after sub-tenon triamcinolone injection
Schröder et al [48]	2015	Case report	Germany	Female	1/1	Macular edema	Treatment was discontinued when ischemic findings appeared
Thoo et al [52]	2014	Case report	Australia	Female	2/2	Macular edema	Patient refused treatment withdrawal and improvement was observed after intravitreal triamcinolone injection
Turaka et al [54]	2012	Case report	USA	Male	1/1	Macular edema	Improvement after treatment withdrawal
Cifuentes-Canorea et al [57]	2019	Case report	Spain	Female	1/1	Macular edema	Improvement after treatment withdrawal and reappearance after restart
Husmann et al [58]	2020	Case report	USA	Female	1/1	Macular edema	Treatment was not withdrawn. Macular edema improved with topical nepafenac
Lapierre et al [63]	2016	Cross-sectional	Canada	Both	11 and 2/2399	Macular edema and uveitis	Improvement after treatment withdrawal
Laroni et al [64]	2016	Cross-sectional	Italy	Both	3/825	Macular edema	2/3 patients withdrew treatment; one remained with macular edema and one improved. In the last patient, treatment was temporarily interrupted and restarted after improvement
Ontaneda et al [67]	2012	Cross-sectional	USA	Both	3/317	Macular edema	Undetermined
Afshar et al [20]	2013	Case series	USA	Both	3/3	Cystoid macular edema	Improvement after treatment withdrawal was seen in two patients. One patient continued fingolimod, with improvement after treatment with nepafenac and diflurprednate
Asensio-Sánchez et al [22]	2014	Case report	Spain	Female	1/1	Cystoid macular edema	No improvement after treatment withdrawal
Fan Gaskin et al [33]	2015	Case report	Australia	Female	1/1	Cystoid macular edema	Improvement after treatment withdrawal, topical diclofenac and topical prednisolone
Pul et al [44]	2016	Case report	Germany	Female	1/1	Cystoid macular edema	Improvement after treatment withdrawal and intravitreal ranibizumab injection
Ueda et al [55]	2015	Case report	Japan	Male	1/1	Cystoid macular edema and retinal hemorrhages	Macular edema was treated with betamethasone after a 13-week persistence and hemorrhages resolved after 24 weeks
Zarbin et al [73]	2013	Cross-sectional	Multicentric	Both	19 and 1/2615	Macular edema and retinal branch vein occlusion	Improvement after treatment withdrawal
Bhatti et al [24]	2013	Case report	USA	Female	1/1	Macular hemorrhage	Improvement after treatment withdrawal
Christopher et al [26]	2017	Case report	USA	Female	1/1	Conjuctival lymphoma	Improvement after treatment withdrawal and rituximab treatment
Gallego-Pinazo et al [32]	2011	Case report	Spain	Female	1/1	Retinal branch vein occlusion	Improvement after treatment withdrawal and intravitreal ranibizumab injection
Lim et al [76]	2019	Cross-Sectional	Multicentric	Both	27/27,528	Uveitis complicated with macular edema	One case required glaucoma topical treatment, two cases oral prednisone and the remaining cases received topical steroid preparations
STEROIDS							
Saatci et al [45]	2010	Case report	Turkey	Male	1/1	Acute retinal necrosis	No improvement after treatment withdrawal. Vitrectomy and photocoagulation were required, and retinal detachment occurred after 2 months
Sheikh et al [49]	2016	Case report	USA	Female	1/1	Acute retinal necrosis	No improvement after treatment withdrawal
CTLA4Ig							
Viglietta et al [17]	2008	Non-RCT	USA	Both	1/20	Visual field defect	Not reported
ESTRIOL							
Voskuhl et al [16]	2016	RCT	USA	Both	4/158	Visual defects (blurred vision or diplopia)	Not reported
INTERFERON-β							
Bakri et al [23]	2015	Case report	USA	Female	1/1	Intraretinal hemorrhages	Treatment was continued under strict follow-up as the patient was asymptomatic
Massougnes et al [28]	2016	Case report	Switzerland	Male	2/2	Retina peripheral bilateral telangiectasiae	Treatment was not withdrawn, and no progression was evidenced
De Santi et al [29]	2005	Case report	Italy	Female	1/1	Sicca syndrome	Treatment was not withdrawn and improvement was observed with oral methylprednisolone treatment
Gaetani et al [31]	2015	Case report	Italy	Female	1/1	Retinopathy	Improvement after treatment withdrawal
Jenisch et al [36]	2012	Case report	Germany	Female	1/1	Branch arterial occlusions and central vein occlusion	Treatment was not withdrawn. Visual acuity improvement was observed after hydroxyethyl- starch and salicylic acid treatment
Longmuir et al [39]	2007	Case report	USA	Male	1/1	Retinopathy	Improvement after treatment temporal suspension
Mallada-Frechín et al [40]	2005	Case report	Spain	Female	1/1	Retinopathy	Improvement after treatment withdrawal
Ohira et al [42]	2009	Case report	Japan	Female	1/1	Retinopathy	Improvement after treatment withdrawal
Post et al [43]	2009	Case report	Canada	Female	1/1	Visual field disturbance	Improvement after treatment withdrawal
Saito et al [46]	2007	Case report	Japan	Male	1/1	Retinopathy	Improvement after treatment withdrawal
Sallansonnet-Froment et al [47]	2009	Case report	France	Male	1/1	Retinopathy	Improvement after treatment withdrawal
Sommer et al [50]	2001	Case report	France	Male	1/1	Retinopathy	Improvement after treatment withdrawal
Spierer et al [51]	2011	Case report	Israel	Female	1/1	Idiopathic orbital inflammation	Treatment was not withdrawn. Symptoms improved with oral prednisolone
Williams et al [60]	2004	Case series	USA	Both	3/3	Neuroretinitis	Not reported
Makioka et al [66]	2017	Cross-sectional	Japan	Both	1/1476	Retinopathy	No improvement after treatment withdrawal
Smith et al [69]	2015	Cross-sectional	USA	Both	Incidence rate of 4.04 / 100 patients per year of 8107 adults	Abnormal vision and xerophthalmia	Undetermined
Tremlett et al [8]	2008	Cross-sectional	Canada	Both	1/888**	Papilledema	Not reported
Liscić et al [65]	2004	Cross-sectional	Croatia	Both	2/9	Abnormal visual evoked potentials	No improvement after treatment withdrawal
Gugliandolo et al [74]	2018	Cross-sectional	Italy (three regions: Liguria, Sicily, and Sardinia)	Both	2/6039	Eyelid edema and visual field defect	Visual field defect resolved within a day
Pakdaman et al [18]	2018	RCT	Iran	Both	Avonex: 3/89 Cinnovez: 1/93	Visual disturbance	Not reported
Comi et al [77]	2019	RCT	Multicentric	Both	Ozanimod 1 mg: 1/447 Ozanimod 0.5 mg: 1/451 Interferon-B1a: 1/448	Macular edema	Not reported
NATALIZUMAB							
Boster et al [25]	2013	Case report	USA	Male	1/1	Progressive Multifocal Leukoencephalopathy by JCV	Patient passed away
Zecca et al [56]	2009	Case report	Switzerland	Male	1/1	Ocular toxoplasmosis reactivation	Treatment withdrawal and treatment with pyrimethamine sulfadiazine, prednisolone and folinic acid with lesion reduction
Holmén et al [62]	2011	Cross-sectional	Sweden	Both	19/85	Ocular inflammation	Not reported
Saida et al [13]	2016	RCT	Japan	Both	1/12	Retinal detachment	Undetermined
HYPERBARIC OXYGEN							
Lambrou et al [38]	1987	Case report	France	Female	1/1	Slow-recovering central bilateral scotoma	Improvement after treatment withdrawal
RITUXIMAB							
Rommer et al [68]	2015	Cross-sectional	Germany	Both	1/56	Ocular inflammation	Not reported
SIPONIMOD							
Selmaj et al [14]	2013	RCT	Multicentric	Both	1/297	Macular edema and optic neuritis	Undetermined
Kappos et al [12]	2018	RCT	Multicentric	Both	18/1099	Macular edema	Not reported
TERIFLUNOMIDE							
Vermersch et al [15]	2013	RCT	Multicentric	Both	Teriflunomide 7 mg: 3/111	Optic neuritis, macular edema, and optic ischemic neuropathy	Not reported
TOVAXIN							
Fox et al [11]	2012	RCT	USA	Both	1/100	Diplopia	Not reported

** This study measured TEAEs, not patients

### Experimental studies

A total of 11 experimental studies were included [9–19], which were published between 2010 and 2019. Six were multicentric across continents, three were conducted in the USA, one was conducted in Japan, and one was conducted in Iran.

After JBIQT qualification, the average number of items marked “Yes” across the studies was 11/13 (range 10–13) for the RCTs, and one study was a nonrandomized controlled trial with 7/9 of the items marked “Yes.” Six studies had a sample size ≤400 patients.

### Observational studies

We collected 56 observational studies, of which 38 were case reports [20–58], four were case series [59–61], and 14 were cross-sectional studies [8, 62–75]. These studies were published between 1987 and 2020. After JBIQT qualification, the average number of items marked “Yes” across the observational studies was 7/8 (range 6–8) for the cross-sectional studies, 7/8 (range 6-8) for case reports, and 9/10 (range 6-10) for case series.

### Adverse events related to each therapy

#### TEAEs related to alemtuzumab

Five studies from Germany, USA, and the United Kingdom (UK) referred to TEAEs when using alemtuzumab, of which three reported the development of thyroid eye disease (TED) [53, 59, 61].

One of the studies was a case series extracted from two clinical trials (CARE-MS1 and CARE-MS2) where five patients developed Graves’ disease (GD), but only two patients had TED [59]. According to Trinh et al [53], there was a female patient who developed bilateral ocular hyperemia, photophobia, proptosis, and upper eyelid retraction, consistent with TED, after three years of treatment [53]. A similar study from the UK reported 10 patients of 162 with GD, but only six patients presented TED, with a period of 26 months, 20 months, four years, 10 months, four years, and 48 months after the last infusion of alemtuzumab, respectively [61].

In a cross-sectional study conducted by Tuohy et al, thyroid autoimmunity developed in 35/87 patients (40.2%) of whom 22/35 (63%) had hyperthyroidism. Although the study reported GD, it was not clear whether the patients had any ophthalmological manifestation. Two patients in that study developed Herpes zoster ophthalmicus [70]. In another study, the use of alemtuzumab was related to thyroid gland autoimmunity (35%), but no TED was reported, and 1% of the patients presented conjunctivitis; however, the study did not specify the etiology of conjunctival inflammation [72].

#### TEAEs related to amantadine

We found two case reports from the USA that described four cases of bilateral corneal edema as a TEAE related to amantadine used for managing tremor and fatigue in patients with MS [30, 35]. The duration of amantadine treatment before the onset of symptoms in all cases ranged from two months to six years, and the range of corneal thickening was 677 μm to >1000 μm [30, 35]. In two cases, the corneal edema resolved approximately one month after amantadine was suspended [30, 35]; in one case, the corneal edema resolved two months after amantadine suspension [35]; and in one case, the corneal edema required management with bilateral penetrating keratoplasty [35].

#### TEAEs related to fingolimod

A total of 25 studies reported ocular TEAEs in patients treated with fingolimod. Of these studies, 18 were case reports, mostly from the USA, Australia, Spain, and Germany; two were experimental studies, both multicentric; and five were cross-sectional studies, one from Canada, one from Italy, one from the USA, and two were multicentric.

Macular edema was reported in 21 studies [9, 10, 20–22, 27, 33, 34, 37, 41, 44, 48, 52, 54, 55, 57, 58, 63, 64, 67, 73]; five were classified as cystoid macular edema [20, 22, 33, 44], and one study reported associated retinal hemorrhages [55]. One study described that the symptoms entirely resolved after the discontinuation of medication; when the medication was restarted after two months, the symptomatology reappeared [57]. One patient had macular edema and retinal branch vein occlusion [73], and one patient had macular hemorrhage [24]. In one case report, fingolimod was not withdrawn, and topical nepafenac was used, which improved the macular edema [58]. Of the remaining three studies, one [26] reported conjunctival lymphoma, one reported retinal branch vein occlusion [32], and one described uveitis complicated with macular edema. In the last study, the authors inferred that the TEAE could not be exclusively attributed to fingolimod because uveitis can by itself cause macular edema [76].

#### TEAEs related to steroids

We found two case reports that described acute retinal necrosis as a TEAE after high-dose steroid treatment for relapsing-remitting multiple sclerosis (RRMS), one from Turkey and the other from the USA [45, 49]. In the first report, the patient received an IV steroid pulse of 1000 mg/day for three days and was continued with 10 mg of daily prednisolone. After two months, the visual symptoms started and the diagnosis of acute retinal necrosis caused by the Varicella-zoster virus was made [45]. In the other case report, the patient received three courses of high-dose methylprednisolone (1000 mg/day for three days) for recurrent relapses five months before the presentation of visual symptoms. The diagnosis of Herpes simplex virus type 2 infection was made [49].

#### TEAEs related to CTLA-4 Ig

This immunoglobulin was used in a Phase 1 clinical trial conducted on 20 patients with RRMS from the USA, which evaluated its safety and tolerability in different doses. This study reported, a visual field defect in one patient receiving a 2 mg/kg dose of the drug within the first 24 h of infusion. During the long-term follow-up, 10% of the enrolled patients had blurred vision [17].

#### TEAEs related to estriol

A Phase 2 clinical trial conducted on patients with RRMS from 16 academic neurology clinics in the USA, evaluating the effect of estriol treatment in the reduction of MS relapses in women, reported TEAEs within 24 months of treatment. There were six events of visual defects (blurred vision or diplopia) reported in four patients receiving estriol compared to seven of the same visual defects reported in seven placebo patients [16].

#### TEAEs related to interferon β

Our review included 21 studies that involved TEAEs after INF therapy, mostly in patients with RRMS.

There were 13 case reports, mostly from Europe (*n* = 7), USA (*n* = 2), Japan (*n* = 2), Canada (*n* = 1), and Israel (*n* = 1). Most of the patients were women (*n* = 8). Patients’ age ranged from 30 to 58 years. The majority of them complained about symptoms after 11 months since the initiation of the treatment. The TEAEs reported in these patients included unilateral/bilateral asymptomatic retinopathy (peripheral intraretinal hemorrhages and cotton wool spots), unilateral/bilateral symptomatic retinopathy (blurred vision, progressive visual field loss, and peripheral telangiectasia), central vein and arterial branch occlusions, Sicca syndrome, and recurrent idiopathic orbital inflammation [23, 28, 29, 31, 36, 39, 40, 42, 43, 46, 47, 50, 51].

One study was a case series that reported three patients with neuroretinitis after INF β therapy, of whom two had been undergoing INF therapy for around a year before the symptoms appeared, the other patient presented with a macular star exudate on funduscopic examination concurrently with the initiation of treatment [60].

There were five cross-sectional studies (one each from Japan, the USA, Canada, Croatia, and Italy). Makioka et al. reported only one case of retinopathy in a postmarketing drug surveillance study for INFβ-1a intramuscular injection in Japan, among a sample of 1441 patients [66]. Smith et al. conducted a postmarketing safety profile study in adult patients with MS with a prescription of INFβ-1a SC three times a week between 2006 and 2012. Some of the patients complained about abnormal vision and xerophthalmia. There were reports of retinal artery or vein obstructions and retinopathy [69]. Tremlett et al. summarized the reported TEAEs for 10 years in Canada in the adult and children population. A 43-year-old female patient presented with papilledema and visual disturbance related to malignant neoplasm aggravation and resulted in death [8]. Liscić et al. evaluated pattern-reversal visual evoked potentials (VEP) in patients with RRMS on INFβ-1a treatment (*n* = 9 patients, 18 eyes). Those with previous optic neuritis (*n* = 3 patients, 3 eyes) exhibited visual evoked potential impairment with a delay in P100 latency before treatment. Moreover, some patients (*n* = 2 patients, 3 eyes) without previous impairment exhibited increased P100 latency after INF therapy. It is not clear whether this impairment is due to INF therapy or MS itself [65]. A multicentric pharmacovigilance study conducted to analyze 10 drugs used for MS treatment over a period of 24 months recorded 411 adverse reactions. Approximately 42% of the TEAEs were due to INF, from which approximately 9% were unexpected, and 5.8% were severe events. Two patients presented ophthalmological manifestations. The first patient, associated with peginterferon-β-1a, with  previous use of dimethyl fumarate, presented mild-moderate eyelid edema. The second patient, associated with glatiramer acetate, with  previous use of INFβ-1a, presented moderate-severe visual field defect, which resolved within a day [74].

Finally, we included two experimental studies. The first one was a double-blind RCT evaluating the comparative efficacy and safety of two different trademarks of INFβ-1a in patients with RRMS conducted by Pakdaman et al. It included 182 participants, of whom 89 received trademark A and 93 trademark B. Regarding TEAEs, 3.4% of participants receiving trademark A and 1.1% receiving trademark B reported visual disturbances [18]. The second study was a multicentric, randomized Phase 3 clinical trial conducted by Comi et al. The aim was to evaluate the safety and efficacy of ozanimod compared with INFβ-1a in patients with RRMS. It enrolled 1346 participants, of whom 447 received 1 mg of ozanimod, 451 received 0.5 mg of ozanimod, and 448 received INFβ-1a. One participant in each treatment group reported a TEAE. The patient in the INFβ-1a group presented macular edema [77].

#### TEAEs related to natalizumab

We found four articles regarding natalizumab [13, 25, 56, 62], including two case reports (one from the USA and the other from Switzerland), one cross-sectional study (from Sweden), and one RCT (from Japan).

Of the two case reports, one [25] reported a case of progressive multifocal leukoencephalopathy (PML) associated with John Cunningham virus (JVC) that started with a subacute onset of bilateral blindness after the 44th dose of natalizumab. The second study reported the first published case of ocular toxoplasmosis reactivation during natalizumab treatment [56].

A web-based MS registry cross-sectional Swedish national postmarketing surveillance study reported 19 patients with TEAEs related to ocular inflammation, herpes simplex, herpes zoster, urinary tract infection, enterovirus meningitis, increased infection susceptibility, and hepatitis C infection. However, the study did not clarify how many of the 19 patients presented the eye compromise. Furthermore, the study reported three cases of PML that occurred during these trial periods, including two cases in the open-label extension phase of the SENTINEL trial and one case in a clinical trial for Crohn’s disease [78, 79]. There was no information regarding the visual compromise in the follow-up of patients who developed PML [62].

The RCT reported a patient with retinal detachment 75 d after the first dose of natalizumab [13].

#### TEAEs related to hyperbaric oxygen

Of the 62 included articles, only one case report published in 1986 described TEAE due to hyperbaric oxygen in a patient with MS who presented slow-recovering central bilateral scotoma [63].

#### TEAEs related to rituximab

One case of ocular inflammation as a TEAE was found after the initiation of treatment, which was reported at the German Registry of autoimmune diseases (GRAID) in a multicentric retrospective study. The time between treatment onset and the TEAE was not determined [68].

#### TEAEs related to siponimod

We found two studies regarding siponimod. One was a multicentric Phase 2 clinical trial conducted on patients with RRMS from 73 specialized MS centers in Canada, the USA, Russia, and nine European countries, wherein the patients were divided into cohort 1 (*n* = 188) and cohort 2 (*n* = 109). It reported that one patient with a history of uveitis presented macular edema with the highest dose of the drug (10 mg), and one patient presented optic neuritis with 0.5-mg dose of the drug [14]. The second study was a Phase 3 clinical trial conducted on patients with secondary progressive multiple sclerosis (SPMS) from 292 hospitals, clinics, and specialized MS centers in 31 countries. It reported that 2% (*n* = 18) of patients receiving siponimod presented macular edema compared to <1% (*n* = 1) of those receiving placebo [12].

#### TEAEs related to teriflunomide

In an experimental study conducted on patients with RRMS from different countries of America, Europe, and Africa, 324 patients were divided into the following three treatment groups: INFβ-1a (*n* = 104), teriflunomide 7 mg (*n* = 109), and teriflunomide 14 mg (*n* = 111). The second group was treated for 66.6 weeks, and the third group was treated for 64.2 weeks. The ocular TEAEs reported were optic neuritis, macular edema, and ischemic optic neuropathy in the second group of patients who received 7 mg of teriflunomide. No ocular TEAEs were observed in groups 1 and 3 [15].

#### TEAEs related to tovaxin

We found an experimental study that evaluated the safety of tovaxin for 24 weeks in patients with RRMS from 30 to 40 different sites in the USA. Neither deaths nor discontinuations due to safety reasons were reported in that study. Diplopia was the only reported ocular event, and it was considered as a major reaction because of the necessity of in-hospital attention of the patient, but not because of the event itself. The authors did not consider this reaction as secondary to the drug and evaluated tovaxin as a safe treatment for the course of 24 weeks [11].

## Discussion

### Methodological assessment of TEAEs

In 2016, PRISMA published an article about harm-reporting in systematic reviews, where terms such as adverse drug reaction, adverse effect, adverse event, complication, harm, side effect, and toxicity were discussed [80]. However, there exists a large heterogeneity when using these terms and reporting TEAEs. This represents a difficulty at the time of reading and interpreting articles about drug safety. Moreover, the diversity between the different analytic and data collection strategies, the different forms of presentation of TEAEs, and the variety among the study designs represented additional challenges in this systematic review.

Two available strategies should be used to record information about TEAEs, i.e., active monitoring, when an event is known or suspected to be associated with an intervention, and spontaneous monitoring for new or unexpected events [81]. Different methodological designs were included in this systematic review because active monitoring generally evaluates long-term TEAEs in RCTs and short-term TEAEs in large cohort studies or case-control studies [81], whereas spontaneous monitoring commonly describes TEAEs in case series and case reports. Each study included in this systematic review had to be carefully evaluated, because spontaneous reports may lead to underreporting and false positives, RCTs may not identify new events, observational studies may require a large sample size, and case reports may not be well documented and have a long-time elapse until publication [82].

### Geographical distribution

Developed countries have a higher incidence of MS. As the populations in these countries have better access to healthcare and early treatment strategies, it is expected that most of the studies that evaluated adverse ophthalmological events - and adverse events in general - had been conducted in these countries. This is consistent with our results, which indicated that studies were largely conducted in Europe, North America, and Japan. A few case reports were described in Australia and Israel. Some multicentric studies included a couple of Asian countries apart from Japan as well. There were no reports of adverse events from other countries or regions, which may result in a poor understanding of the entire picture worldwide.

### Pregnancy and hormones in MS

MS relapses are decreased during pregnancy (primarily during the third trimester) and influenced by sex hormones such as estriol [16]. One RCT demonstrated that estriol was related to visual defects (blurred or double vision); nonetheless, the same visual defects were reported in the placebo group. In clinically isolated syndromes, the incidence of some of the typical features such as visual acuity and color vision was reduced [83]; therefore, the findings might not be directly related to estriol-associated TEAEs. These manifestations have also been described in acute optic neuritis, in which patients described blurred vision [84]. Furthermore, optic neuritis was related to different MS treatments such as INFβ-1b, siponimod, and teriflunomide [14, 15, 85]. However, the resolution of TEAEs after medication withdrawal was not described.

### Follow-up recommendations

Optic neuritis is one of the primary manifestations of MS; therefore, ophthalmological follow-up is mandatory in these patients. Complete ophthalmological examination with attention to the efferent visual system and ancillary studies such as VEP and optic nerve optical coherence tomography (OCT) should be available for these patients [86].

Based on the present study, fingolimod-related TEAEs were highly common. We suggest the following American Academy of Ophthalmology recommendations for patients with MS being treated with fingolimod published in October 2011: 1) A screening evaluation for pre-existing uveitis or macular or retinal vascular disease before starting, or within the first few weeks of starting fingolimod. 2) A single re-evaluation at 3–4 months of therapy. 3) Patients should be advised that the incidence of macular edema is low (~2/1000), but if there is a history of uveitis, the incidence may be as high as 20%. 4) Visual acuity check and a complete eye exam, including a dilated fundus exam, is a proper ophthalmic screening protocol. 5) Patients with abnormalities on the exam or unexplained decreased visual acuity might benefit from diagnostic imaging with macular OCT [87]. These recommendations are supported by the methodology used in the study conducted by Zarbin et al. in 2013, who reported differences in different times of evaluation of these patients [73].

Regarding the use of alemtuzumab in patients with MS and thyroid disease, it was reported that up to 22% of patients treated with alemtuzumab would develop GD [53]. In almost 90% of the cases, GD and Graves ophthalmopathy (GO) co-occur; nevertheless, GO can develop after up to 1 year from being diagnosed with GD [88]. Therefore, we suggest an annual check-up by ophthalmologists, neurologists, and endocrinologists. This follow-up is important to ensure that patients undergo timely ophthalmological treatment while simultaneously ensuring the continued success of alemtuzumab treatment in the management of MS [53, 59, 70].

In contrast, one of the studies on patients treated with alemtuzumab reported Herpes zoster ophthalmicus infection [70]. Previous trials have identified Herpes simplex virus and Varicella-zoster virus systemic opportunistic infections are among the most common infections in patients treated with alemtuzumab and recommended preventive measures, including antiherpetic prophylaxis [89].

Studies evaluating the presence of corneal edema secondary to the use of amantadine in patients with MS corresponded to case reports. Considering that corneal findings can occur from two months to six years after medication initiation, we recommend that the neurologist treating the patient be aware of the warning signs to immediately consult the ophthalmology specialist and prevent irreversible endothelial failure [30, 35].

Another study evaluated the presence of dry eye after the use of anticholinergics, based on which we recommend that the neurologist treating patients with MS must be aware of this TEAE. Although the symptoms should not be the reason to stop an efficient treatment, it is recommended to conduct an ophthalmological evaluation to treat dry eye [71].

The study that evaluated CTLA-4 Ig treatment in patients with MS demonstrated  blurred vision in 10% of the patients. However, the cause of blurred vision was not clear, and the spectrum of MS manifestations included blurred vision secondary to optic neuritis and uveitis. This is why further studies are required to evaluate the safety of this drug in patients with MS, assess whether the ocular compromise corresponds to a TEAE, and generate recommendations on this issue [17].

The clinical trial comparing estriol combined with glatiramer acetate and placebo for women with RRMS reported six blurry and double vision events after six months of follow-up. However, the study did not report the etiology of the visual problems, and hence it is not possible to know whether it corresponds to the natural course of the disease. Further studies are necessary to evaluate the safety of this medication [16].

Two case reports described acute retinal necrosis caused by HSV-2 and Varicella-zoster virus in patients undergoing high-dose steroid therapy [45, 49]. Therefore, we recommend a retina specialist evaluation for patients showing ocular symptoms such as floaters, blurred vision, and sudden vision loss after treatment with steroid pulses.

Patients undergoing INFβ-1a therapy showed multiple ocular TEAEs in a duration range of three months to 10 years (Table 1). Given the wide timeframe for the establishment of TEAEs, and that some of the pathologies may correspond to manifestations or associations of MS, we consider that the most appropriate recommendation for these patients is the evaluation by an ophthalmologist before starting treatment and at least once a year after the start of treatment.

We consider that there is insufficient information to provide screening recommendations regarding natalizumab, hyperbaric oxygen, rituximab, siponimod, teriflunomide, and tovaxin. Nevertheless, in the presence of any ocular symptoms, we strongly recommend an immediate evaluation by an ophthalmology specialist.

Table 2 shows the referral recommendations for each TEAE reported in this systematic review, based on our clinical experience. However, it is important to consider that each patient's case should be individualized, and disease guidelines should be followed.

**Table 2:** Referral recommendations for each TEAE

TEAEs	Referral
Papilledema	Neurologist, priority attention
Conjunctivitis	Ophthalmologist
Herpes zoster ophtalmicus	Ophthalmologist and refer depending on the compromise
Visual disturbance	
Ocular inflammation	
Macular edema	Ophthalmologist, if available retina specialist
Retinal branch vein occlusion	
Macular hemorrhage	
Retinal hemorrhages and macular edema	
Retinopathy	
Intraretinal hemorrhages	
Retina peripheral bilateral telangiectasiae	
Neuroretinitis	
Retinal detachment	
Acute retinal necrosis	
Ocular toxoplasmosis reactivation	Ophthalmologist, if available retina or uvea specialist
Uveitis	Ophthalmologist, if available uvea specialist
Abnormal visual evoked potentials	Ophthalmologist, if available neuro-ophthalmologist
Visual field defect	
Progressive multifocal leukoencephalopathy by JCV	
Optic ischemic neuropathy	
Diplopia	
Thyroid eye disease	Endocrinologist and ophthalmologist, if available oculoplastics specialist
Eyelid edema	Ophthalmologist, if available oculoplastics specialist
Conjuctival lymphoma	
Idiopathic orbital inflammation	Ophthalmologist, if available ocular surface specialist
Sjogren syndrome	Rheumatologist and ophthalmologist, if available ocular surface specialist
Corneal edema	Ophthalmologist, if available cornea specialist

## Conclusions

Considering that ophthalmological TEAEs are generally undermined in clinical trials, different methodological designs were included in this systematic review. In light of this, it is necessary to conduct longitudinal studies to provide evidence-based recommendations.

Despite the diverse geographical distribution of the studies, there were scarce data from Latin America and African countries. Therefore, we strongly encourage healthcare providers to conduct pilot studies in these regions.

We suggest that physicians perform a comprehensive interrogatory and evaluation for ocular signs and symptoms during the follow-up of patients receiving treatment for MS and refer when necessary. An interdisciplinary approach might be considered to evaluate the patient’s requirements.

## Supplementary Information

**Additional file 1.** : Annex 1

**Additional file 2.** : Annex 2

## Data Availability

The datasets used and/or analyzed during the current study are available by the corresponding author on reasonable request.

## Abbreviations

ADLT: Alejandra de-la-Torre
DeCS: Descriptores en Ciencias de la Salud
EZB: Estefanía Zapata-Bravo
Emtree: EMBASE subject headings
GRAID: German Registry of Autoimmune Diseases
GD: Graves’ disease
GO: Graves ophthalmopathy
HSV: Herpes simplex virus
INF: Interferon
JMO: Juliana Muñoz-Ortiz
JRG: Juliana Reyes-Guanes
JARH: Juan Antonio Reyes-Hurtado
JBIQT: Joanna Briggs Institute Quality Tool
JVC: John Cunningham virus
LMM: Laura Mora-Muñoz
LOTG: Luis Octavio Tierradentro-García
LILACS: Literatura latinoamericana y del Caribe en Ciencias de la Salud
MS: Multiple sclerosis
MeSH: Medical Subject Headings
MGS: Marcela Gómez-Suarez
MRI: Magnetic Resonance Imaging
PRISMA: Preferred Reporting Items for Systematic Reviews and Meta-Analysis
PML: Progressive multifocal leukoencephalopathy
RRMS: Relapsing-remitting multiple sclerosis
RCT: Randomized controlled trials
SC: Subcutaneous
TEAEs: Treatment-emergent adverse events
USA: United States of America
UK: United Kingdom
VEP: Visual evoked potentials
VZV: Varicella-zoster virus
WRC: William Rojas-Carabali
