# Supplementary material for: Ocular adverse events from pharmacological treatment in patients with multiple sclerosis—A systematic review of the literature
Source: Syst Rev. 2021 Oct 28;10:280. doi: 10.1186/s13643-021-01782-7 (PMC8554884; doi:10.1186/s13643-021-01782-7)
Supplement: Supplementary file 1 — Additional file 1. : Annex 1 [file 13643_2021_1782_MOESM1_ESM.docx]

| **Database** | **Search strategy** |
| --- | --- |
| PUBMED: 1756 | (((((((((multiple sclerosis[MeSH Terms]) OR Sclerosis, Multiple) OR Sclerosis, Disseminated) OR Disseminated Sclerosis) OR MS (Multiple Sclerosis)) OR Multiple Sclerosis, Acute Fulminating) AND Humans[Mesh])) AND (((((((Therapeutics[MeSH Terms]) OR Therapeutic) OR Therapy) OR Therapies) OR Treatment) OR Treatments) AND Humans[Mesh])) AND (((((eye diseases[MeSH Terms]) OR Disease, Eye) OR Diseases, Eye) OR Eye Disease) AND Humans[Mesh]) |
| LILACS: 0 | Esclerosis multiple AND Tratamiento farmacológico AND oftalmopatia (decs) |
| EMBASE: 1093 | 'multiple sclerosis'/exp AND ('pharmacology'/exp OR 'therapy'/exp) AND 'adverse event'/exp AND 'eye disease'/exp |
| COCHRANE: 3 | MeSH descriptor: [Multiple Sclerosis] explode all trees and with qualifier(s): [therapy - TH] AND MeSH descriptor: [Eye Diseases] explode all trees |
| Update from 23/03/20 to 04/05/20 | Pubmed 2  Embase 5  Lilacs 0  Cochrane 0 |
