# Supplementary material for: Ocular adverse events from pharmacological treatment in patients with multiple sclerosis—A systematic review of the literature
Source: Syst Rev. 2021 Oct 28;10:280. doi: 10.1186/s13643-021-01782-7 (PMC8554884; doi:10.1186/s13643-021-01782-7)
Supplement: Supplementary file 2 — Additional file 2. : Annex 2 [file 13643_2021_1782_MOESM2_ESM.pdf]

THE JOANNA BRIGGS INSTITUTE

JBI Critical Appraisal Checklist for Case Reports

Reviewer_____________________________________Date___________________________

Author _____________________________________Year_________Record Number______

		Yes	No	Unclear	Not applicable
1.	Were patient's demographic characteristics clearly described?	✓	☐	☐	☐
2.	Was the patient's history clearly described and presented as a timeline?	✓	☐	☐	☐
3.	Was the current clinical condition of the patient on presentation clearly described?	✓	☐	☐	☐
4.	Were diagnostic tests or assessment methods and the results clearly described?	✓	☐	☐	☐
5.	Was the intervention(s) or treatment procedure(s) clearly described?	✓	☐	☐	☐
6.	Was the post-intervention clinical condition clearly described?	✓	☐	☐	☐
7.	Were adverse events (harms) or unanticipated events identified and described?	✓	☐	☐	☐
8.	Does the case report provide takeaway lessons?	✓	☐	☐	☐

Overall appraisal: Include ☐ Exclude ☐ Seek further info ☐

Comments (Including reason for exclusion)

Major criteria: ✓

Minor criteria: ✓

Inclusion criteria: 5/8 (At least 4 must be major inclusion criteria ✓)

THE JOANNA BRIGGS INSTITUTE

JBI Critical Appraisal Checklist for Case Series

Reviewer_____________________________________Date___________________________

Author _____________________________________Year_________Record Number______

	Yes	No	Unclear	Not applicable
1. Were there clear criteria for inclusion in the case series?	☑	☐	☐	☐
2. Was the condition measured in a standard, reliable way for all participants included in the case series?	☑	☐	☐	☐
3. Were valid methods used for identification of the condition for all participants included in the case series?	☑	☐	☐	☐
4. Did the case series have consecutive inclusion of participants?	☑	☐	☐	☐
5. Did the case series have complete inclusion of participants?	☑	☐	☐	☐
6. Was there clear reporting of the demographics of the participants in the study?	☑	☐	☐	☐
7. Was there clear reporting of clinical information of the participants?	☑	☐	☐	☐
8. Were the outcomes or follow up results of cases clearly reported?	☑	☐	☐	☐
9. Was there clear reporting of the presenting site(s)/clinic(s) demographic information?	☑	☐	☐	☐
10. Was statistical analysis appropriate?	☑	☐	☐	☐

Overall appraisal: Include ☐ Exclude ☐ Seek further info ☐

Comments (Including reason for exclusion)

Major criteria: ✓

Minor criteria: ✓

Inclusion criteria: 6/10 (At least 5 must be major inclusion criteria✓)

© Joanna Briggs Institute 2017

THE JOANNA BRIGGS INSTITUTE

JBI Critical Appraisal Checklist for Analytical Cross Sectional Studies

Reviewer______________________________________Date___________________________

Author ______________________________________Year_________Record Number______

	Yes	No	Unclear	Not applicable
1. Were the criteria for inclusion in the sample clearly defined?	☑	☐	☐	☐
2. Were the study subjects and the setting described in detail?	☑	☐	☐	☐
3. Was the exposure measured in a valid and reliable way?	☑	☐	☐	☐
4. Were objective, standard criteria used for measurement of the condition?	☑	☐	☐	☐
5. Were confounding factors identified?	☑	☐	☐	☐
6. Were strategies to deal with confounding factors stated?	☑	☐	☐	☐
7. Were the outcomes measured in a valid and reliable way?	☑	☐	☐	☐
8. Was appropriate statistical analysis used?	☑	☐	☐	☐

Overall appraisal: Include ☐ Exclude ☐ Seek further info ☐

Comments (Including reason for exclusion)

Major criteria: ✓

Minor criteria: ✓

Inclusion criteria: 6/8 (At least 5 meet the major inclusion criteria✓)

© Joanna Briggs Institute 2017

THE JOANNA BRIGGS INSTITUTE

JBI Critical Appraisal Checklist for Randomized Controlled Trials

Reviewer_____________________________________Date____________________________________

Author ______________________________________Year_______________Record Number______

		Yes	No	Unclear	NA
1.	Was true randomization used for assignment of participants to treatment groups?	☑	☐	☐	☐
2.	Was allocation to treatment groups concealed?	☑	☐	☐	☐
3.	Were treatment groups similar at the baseline?	☑	☐	☐	☐
4.	Were participants blind to treatment assignment?	☑	☐	☐	☐
5.	Were those delivering treatment blind to treatment assignment?	☑	☐	☐	☐
6.	Were outcomes assessors blind to treatment assignment?	☑	☐	☐	☐
7.	Were treatment groups treated identically other than the intervention of interest?	☑	☐	☐	☐
8.	Was follow up complete and if not, were differences between groups in terms of their follow up adequately described and analyzed?	☑	☐	☐	☐
9.	Were participants analyzed in the groups to which they were randomized?	☑	☐	☐	☐
10.	Were outcomes measured in the same way for treatment groups?	☑	☐	☐	☐
11.	Were outcomes measured in a reliable way?	☑	☐	☐	☐
12.	Was appropriate statistical analysis used?	☑	☐	☐	☐
13.	Was the trial design appropriate, and any deviations from the standard RCT design (individual randomization, parallel groups) accounted for in the conduct and analysis of the trial?	☑	☐	☐	☐

Overall appraisal: Include ☐ Exclude ☐ Seek further info ☐

Comments (Including reason for exclusion)

Major criteria: ✓

Minor criteria: ✓

Inclusion criteria: 10/13 (At least 10 must be major inclusion criteria ✓)

THE JOANNA BRIGGS INSTITUTE

JBI Critical Appraisal Checklist for Quasi-Experimental Studies (non-randomized experimental studies)

Reviewer_____________________________________Date__________________________________

Author _____________________________________Year______________Record Number______

		Yes	No	Unclear	Not applicable
1.	Is it clear in the study what is the 'cause' and what is the 'effect' (i.e. there is no confusion about which variable comes first)?	☑	☐	☐	☐
2.	Were the participants included in any comparisons similar?	☑	☐	☐	☐
3.	Were the participants included in any comparisons receiving similar treatment/care, other than the exposure or intervention of interest?	☑	☐	☐	☐
4.	Was there a control group?	☑	☐	☐	☐
5.	Were there multiple measurements of the outcome both pre and post the intervention/exposure?	☑	☐	☐	☐
6.	Was follow up complete and if not, were differences between groups in terms of their follow up adequately described and analyzed?	☑	☐	☐	☐
7.	Were the outcomes of participants included in any comparisons measured in the same way?	☑	☐	☐	☐
8.	Were outcomes measured in a reliable way?	☑	☐	☐	☐
9.	Was appropriate statistical analysis used?	☑	☐	☐	☐

Overall appraisal: Include ☐ Exclude ☐ Seek further info ☐

Comments (Including reason for exclusion)

Major criteria: ✓

Minor criteria: ✓

Inclusion criteria: 6/9 (At least 5 must be major inclusion criteria ✓)
